# Implementing a Clinical Research Department to Support Pediatric Studies: A SWOT Analysis

**DOI:** 10.3390/ijerph17176211

**Published:** 2020-08-27

**Authors:** Alexandra Thajer, Margit Sommersguter-Reichmann, Henriette Löffler-Stastka

**Affiliations:** 1 Department of Pediatrics and Adolescent Medicine, Medical University of Vienna, 1090 Vienna, Austria; alexandra.thajer@meduniwien.ac.at; 2 Department of Finance, Karl-Franzens University Graz, 8010 Graz, Austria; 3 Department of Psychoanalysis and Psychotherapy, Medical University of Vienna, 1090 Vienna, Austria; henriette.loeffler-stastka@meduniwien.ac.at

**Keywords:** SWOT analysis, pediatric studies, company analysis, environmental analysis, general environment, task environment

## Abstract

The safety, tolerability, pharmacokinetics and efficacy of most drugs used in pediatrics have not been studied in different age groups and are administered “off-label use”. Clinical pediatric drug trials require specific and stringent compliance with laws, regulations, guidelines, and patient/parent/public involvement, which in turn increases resource use and makes support useful from a medical, qualitative, economic, and system perspective. We examined the strengths, weaknesses, opportunities and threats of implementing a Research Department for the Support of Pediatric Studies (RDPS) in Vienna. We used the SWOT (“strengths”, “weaknesses”, “opportunities”, and “threats”) analysis to collect comprehensive data and facts on the internal strengths, weaknesses (company analysis), and external opportunities and threats (environmental analysis). The company analysis revealed a productivity gain, due to a highly specialized team and standardized processes. The environmental analysis outlined a considerable 360-degree potential for a qualitative and quantitative medical- and social-scientific expansion of the service portfolio. The establishment of a RDPS leads to the centralization of pediatric studies by bundling tasks and concentration of specialist knowledge, which enables the exploitation of synergies, the standardization of processes, the promotion of professionalism, flexibility, innovations and the reduction of inefficiencies in the form of duplication of tasks. RDPS offers tailored advice and support for different types of pediatric studies.

## 1. Introduction

The demands on healthcare facilities and their decision-makers are continuously increasing. Decision-makers of all medical disciplines have to make and constantly adapt medical and economic decisions in order to achieve or maintain optimal performance in a dynamically changing environment.

Clinical research is an important pillar of evidence-based medicine, which combines individual clinical expertise with the best available external clinical evidence from systematic research to support medical decisions. The individual clinical expertise encompasses the competence of the clinician, while the external clinical evidence processes clinically relevant and patient-centered research results [1]. Clinical research therefore plays an important role with regard to the request for standardized clinical decision-making.

Researchers already have pointed out avoidable weaknesses in clinical research in general [2]. These include the suboptimal composition of research staff, the inadequate training of clinical researchers and laboratory assistants, the inadequate documentation of research decisions and the lack of reproducibility of the results, which jeopardize the quality of the findings and lead to a considerable waste of resources.

In pediatrics, clinical research even lags behind the clinical research involving adults. In the field of pediatrics, decision-makers face the problem that the safety, tolerability, pharmacokinetics and efficacy of most medicinal products have not been systematically tested on different age groups of the pediatric patient population [3], which includes children and adolescents between birth and the age of 18 [4]. Klassen et al. emphasized that, “…adapting adult evidence to children can result in ineffective or even unsafe medical care” [5]. For example, children and adolescents receive medication “off-label use”, i.e., beyond the approval of the pharmaceutical authorities, in around 42–90 percent in hospital care and 46–64 percent in outpatient care. In neonatology, more than 90 percent of drugs are administered in off-label use [6,7,8]. The off-label use of drugs can influence age groups, indication, dosage or type of use (e.g., pills, capsules, solutions for injection, suppositories and syrups) [9]. A review of Martinez-Castaldi et al. also revealed several deficiencies of clinical research involving the pediatric population that have the potential to endanger the quality of care for children [10].

In clinical drug trials for the pediatric population, compliance with manifold laws, regulations, and guidelines is required [3], which in turn increases resource use. In addition, there is also a need for medical device studies, non-interventional drug studies, registry studies and other studies (e.g., epidemiological studies) in the pediatric field, which will significantly increase the demand for support of such studies.

Further, this population often requires a careful patient/public involvement and participatory research design in all kinds of studies, in either clinical trials or observational studies including a mixed methods design (including qualitative data collection). Patient/parent preferences and stakeholder priorities have to be considered to ensure user compliance and system-level financial obligations and policies. Dissemination strategies (focus groups, panels with professionals, stakeholder meetings) have to be calculated. For appropriate clinical reasoning and clinical decision-making, the entire system and its interaction processes must be taken into account [11,12]. With the increasing number of studies at the Department of Pediatrics and Adolescent Medicine (UKKJ) at the Medical University in Vienna in recent years, the demand for support has also increased; not least, because the workload to conduct pediatric clinical trials is very high due to the very strict national and international legal and regulatory requirements.

In view of these challenges and the expected increase in pediatric studies, we investigated the role of a Research Department for the Support of Pediatric Studies (RDPS) at the UKKJ of the Medical University of Vienna in improving the efficiency and the effectiveness of pediatric research. Put differently, the role a RDPS plays in supporting clinical trials in the pediatric field needs to be carefully assessed in order to evaluate to what extent a RDPS can strengthen all types of research in the pediatric field.

Croghan et al. showed how a department to support clinical trials could be integrated and established at an academic institution in the United States [13]. However, their findings cannot be translated one-to-one with respect to the implementation of a RDPS, because the guidelines and laws for clinical trials differ between the United States and Europe. In addition, pediatric studies with their special requirements were not included in this study. This was a further motivation to investigate the establishment of a department to support studies in children and adolescents only.

Overall, against the background of the above-mentioned shortcomings, we investigated whether the establishment of a novel pediatric research department could increase productivity, effectiveness and research performance in conducting pediatric clinical trials while maintaining or even increasing quality.

For this purpose, we used SWOT analysis to provide decision-makers with a valid basis regarding the pros and cons of implementing a RDPS. SWOT analysis has been widely used in different contexts and industries. Helms and Nixon reviewed 142 SWOT analyses published between 1999 and 2009 in order to categorize the various areas of applications and provide guidance for improving the method and supporting theory building [14]. The authors also reported on the use of SWOT analyses in the healthcare industry, which covered individual facilities such as hospitals as well as entire sectors and industries (e.g., nursing sector and pharmaceutical industry) [14]. The use of SWOT analyses in the pediatric field is also not new. Recent applications include assessing parents’ preferences for improving parental collaboration and compliance with post-discharge health promotion services for preterm born children less than 1250 g at birth [15], as well as assessing reorganization of service delivery in pediatric rehabilitation [16], evaluating the contribution of the media to the promotion of breastfeeding in Mexico [17]; and identifying hearing impairments among children in Italy [18].

By focusing the challenging field of upcoming trials with participatory design, a RDPS also has to foster economic evaluations. The decision as to which diagnostic or therapeutic procedure has to be implemented based on evidence and tested prior to implementation, requires a complex analysis in order to answer the adequate questions in the correct order. Optimizing health care decisions often requires the ability to carry out cost-effectiveness analyses, cost-benefit analyses, cost-consequences analyses or even cost-minimization-analyses easily. Therefore, economic knowledge is also necessary to establish a research department such as the RDPS. Including economic expertise from the very beginning of a strategic planning phase of such a research department is rare.

## 2. Materials and Methods

The SWOT analysis is a simple but efficient management tool to evaluate the strategic positioning of an organization. “SWOT” is the acronym of the terms “strengths”, “weaknesses”, “opportunities”, and “threats”. While strengths and weaknesses are assessed as part of the company analysis, opportunities and threats are gathered in the course of the environmental analysis. Strengths and weaknesses represent company-specific competitive advantages (e.g., provision of innovative products or services, availability of adequate/unique technology, low overhead) and disadvantages (e.g., limited funding capacity, small number of customers or low customer growth, outdated technology). Opportunities and threats represent factors in the company environment, which can be advantageous (e.g., emergency of new markets, positive developments in society, government funding/support) or disadvantageous (e.g., market shrinkage, legal restrictions and requirements, new competitors) [19,20].

SWOT analysis consolidates the results of the company (internal) and environmental (external) analysis in the form of a SWOT matrix, thereby acknowledging the fact that an organization cannot operate in isolation but is in constant interaction with a dynamically changing environment. The SWOT matrix is the basis for prioritizing fields of action and deriving strategies with regard to a particular business alternative [19,20,21,22,23,24].

We used the strategic management approach for the company analysis. With this approach, the company is divided into a management sub-system, including the tasks planning, control, information management, organization, corporate culture, and a service provision sub-system, comprising the tasks of technology, service production in the narrow sense, personnel, and capital. Strategic success factors are then determined for each task [23].

The company environment is analyzed as part of the environmental analysis. With regard to the proximity to the company, we distinguish between the general environment and the task environment. The general environment covers aspects of the population, society, technology, politics, and the entire economy. The task environment refers to the relevant market. The ultimate goal of the environmental analysis is to identify trends in both environments. For this purpose, we used the outside-in approach, which takes the perspective of the environment on the company for the identification of trends [22].

Hill and Westbrook identified three broad approaches to perform a SWOT analysis [25]. First, a single person, who can either be an experienced employee in the company or an external consultant, performs the SWOT analysis. Second, several company executives conduct a SWOT analysis separately and then consolidate their results. Third, the SWOT analysis is the result of meetings of managers all of whom contribute to the results.

This paper used the first approach. The first author of this study, Alexandra Thajer, carried out the SWOT analysis. As a senior researcher, she has relevant expert knowledge in the implementation, coordination and support of pediatric studies. She has also been involved in the establishment and ongoing operation of a pediatric research network. This gives her a tremendous amount of knowledge about what it takes to implement a RDPS. The usefulness of establishing a RDPS to assist physicians in conducting all types of pediatric studies was therefore based on the systematic collection of information in conducting and supporting pediatric studies over a ten-year period. This also included assessing the needs of all parties involved, reviewing and continuously updating legal and regulatory requirements, and analyzing secondary sources such as research articles, websites and government reports.

While some authors criticized the use of SWOT analysis in the healthcare sector [26], we motivated the use of SWOT analysis as a tool to prepare a decision about the future direction of conducting pediatric studies as follows. With the help of the SWOT analysis, relevant internal processes and stakeholders as well as current and future environmental factors are systematically identified and assessed. All of these factors can significantly influence the success of a business strategy, such as the establishment of a RDPS.

SWOT analysis was used to evaluate the implementation of a RDPS at the UKKJ of the Medical University of Vienna, where about 100 feasibility checks from 25 industrial partners and 23 indication areas were carried out over the last six years. Finally, almost 70 studies, mainly pharmaceutical studies, but also medical device studies and registry studies, were conducted. In addition, almost 70 academic studies were performed covering the whole spectrum of clinical trials, from medical products and device studies, non-interventional drug studies, registry studies to other studies and screenings.

## 3. Results

This section summarizes the results of the company and environmental analysis, which form the basis for identifying the strengths and weaknesses as well as opportunities and threats, including their comparison in the form of the SWOT matrix.

### 3.1. Company Analysis

In addition to the organizational structure of the UKKJ and the planned RDPS, we also present information on the different stages of a clinical study and the stage-specific involvement of various factors as a result of the company analysis.

**Figure 1:**
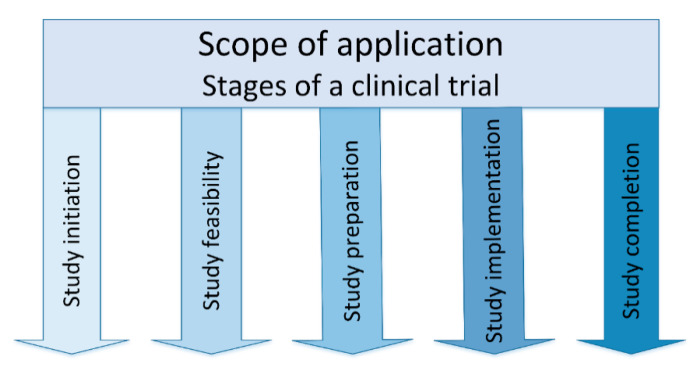
RDPS scope of application—study stages.

A clinical study passes five stages: initiation, feasibility, preparation, implementation and completion (Figure 1).

- *Study initiation:* In the stage of study initiation, a sponsor approaches potential investigators with the question of whether there is interest in conducting a study.
- *Study feasibility:* The feasibility of the planned clinical study is checked and evaluated in the next stage. The sponsor provides a synopsis of the study and the key facts, such as study title, objectives, study design, inclusion and exclusion criteria for the study patients, treatment, investigational product, study procedures, sample size and duration of study. The feasibility of the study, including the site feasibility, is evaluated using a questionnaire.
- *Study preparation:* The study preparation stage is very time-consuming and resource-intensive. The entire study team is determined on site and the study is reported to the local ethics committee (EC), the national competent authority (NCA) and the hospital management. The clinical trial agreement (CTA) and the patient insurance are concluded. The study team must attend Good Clinical Practice (GCP) [27,28] and randomization training, as well as training in how to handle the investigational medicinal product (IMP), including storage, that is usually provided online by the sponsor. In the study preparation stage, the investigational medicinal products including the necessary study materials, such as files, informed consent forms (ICFs), logs, laboratory materials, are sent to the study center.
- *Study implementation:* The study begins after all preparations are complete. The patient comes to the clinic for the study visits according to the clinical trial protocol and receives all study-related examinations and the investigational product (IP). The implementation stage starts with the initiation visit (IV), which is followed by interim (regular/routine monitoring) visits (RMV) [29].
  Once the first patient has been included in the study, the monitoring visits typically take place within two weeks and then every four to eight weeks. During these visits, the source documents and compliance with the study protocol and the GCP guidelines are checked [27,28].
- *Study completion:* The final stage is the completion of the study with the last monitoring visit, the so-called close out visit (COV) [29]. The end of the study must be reported to the ethics committee, the responsible national competent authority and the hospital management.

The implementation of the research department requires the availability of suitable framework conditions, the choice of an efficient department structure and the careful embedding of the department in the existing structure of the organization.

**Figure 2:**
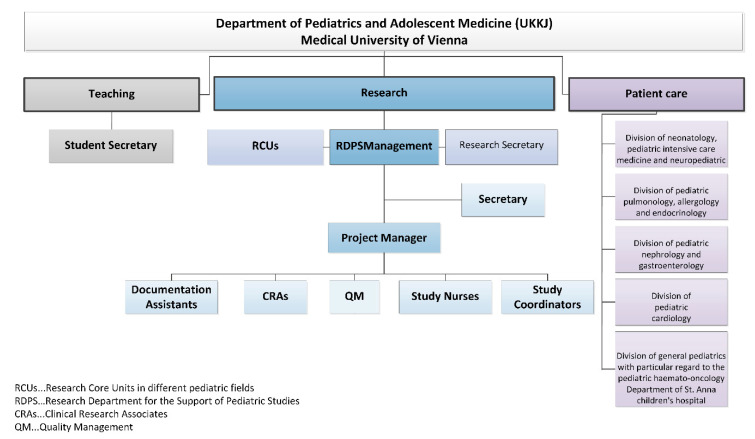
RDPS organizational chart embedded into UKKJ.

The Medical University of Vienna has the following three cornerstones: teaching, patient care and research. With regard to the internal support and promotion of research, only a research secretary and the Research Core Units (RCUs) have so far been embedded in the organizational structure of the UKKJ. The RCUs, which consist of different scientific working groups from different pediatric fields, must be clearly distinguished from the RDPS. The RDPS is a service provider for the RCUs that are to be supported in the implementation of clinical pediatric studies. Although the UKKJ is a large study center, this type of support is not yet available to the RCUs, which means that each working group has to organize the entire study process individually. In addition to patient care, this has proven to be quite a challenge, especially as the current submission requirements from the national competent authority, including regulatory guidelines, monitoring, application of EudcraCT number, etc. are not well known. Figure 2 illustrates the organizational chart of the RDPS. The OKIDS research network and the Clinical Trials Coordination Center (KKS) of Vienna, however, also support the UKKJ externally in order to fulfill its research tasks. OKIDS, a cooperation of pediatric university clinics of various medical universities, was founded in 2013 with the aim of promoting and supporting the implementation of pediatric drug studies. The network for coordination centers for clinical studies (KKS) provides that the individual coordination centers be set up directly at the medical universities. As the KKS Vienna focuses on adult studies it refers to the pediatric module OKIDS by providing a contact person for the support of pediatric studies. The significance of these cooperation partners for the RDPS is explained as part of the SWOT analysis in Section 3.3.

- *RDPS management:* In addition to staff and cost responsibility, RDPS management is also responsible for the promotion of employees and the study development and management.
- *Secretary:* The secretary provides administrative support to management.
- *Project manager:* The project manager (PM) represents the interface between RDPS management and other employees. The main tasks of the project manager comprise communication, advice (initiation and feasibility of studies), support (conduct of studies), review (study documents) and management. Management tasks comprise support of pharmacovigilance and evaluation of resources, budget, study contract, standardized operation procedures and monitoring plans.
- *Documentation assistants:* The documentation assistants are responsible for the entire documentation process of patient records in paper form, the collection of patient data from the archive or from the electronic system of the Vienna General Hospital. The Vienna General Hospital is the public hospital with which the Medical University of Vienna cooperates in performing their clinical research, patient care and teaching tasks. The documentation assistants are in charge of data entry into the study-related (electronic) case report forms (CRFs).
- *Study coordinators:* The study coordinators are responsible for the overall communication and coordination in all stages of a study. They represent the interface between the sponsor and the entire study team. The study coordinators support the completion of feasibility questionnaires, the process of drafting contracts and compliance with regulatory requirements and are responsible for shipping inspection, documentation, archiving, communication with suppliers, to name just a few.
- *Study nurses:* A study nurse plays a key role in conducting clinical trials and represents an important interface between the monitor and the study team. The focus is on the activities with, on and for the patient and everything related to the investigational medicinal product.
- *Clinical research associates:* The clinical research associate (CRA) is responsible for quality assurance and developing a monitoring plan for each clinical trial. The preparation, implementation and follow-up of pre-study visits, initiation visits, routine monitoring visits and close-out visits is the responsibility of a clinical research associate. Other tasks include monitoring of the investigator site file (ISF) and the trial master file (TMF), regulatory affairs, source data verification, data quality verification, patient insurance, laboratory, pharmacy and documenting adverse events. The monitor represents the interface between the sponsor and the study team.
- *Quality management:* With quality management (QM), all activities within the RDPS should be coordinated in such a way that quality can be ensured, checked and if necessary improved and that quality objectives can be met.

The clear and lean departmental structure including clearly assigned tasks for the RDPS staff and a quality management system helps to ensure high-quality clinical trials [30,31].

### 3.2. Environmental Analysis

As result of the environmental analysis we present information about the general and the task environment [22].

#### 3.2.1. General Environment

**Figure 3:**
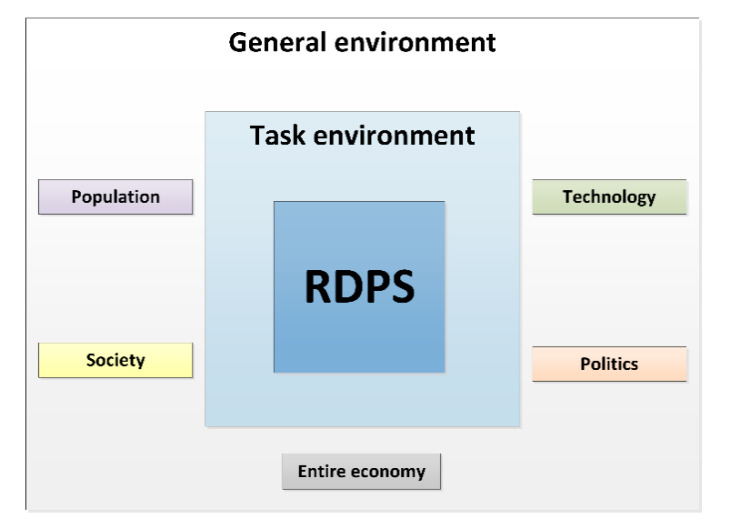
RDPS environmental analysis.

The general environment of a RDPS comprises population, society, technology, politics and the entire economy (Figure 3).

- *Population:* Demographic factors, such as the availability of the pediatric study sample, are relevant for the selection as a study center. The population growth, which can also be attributed to migration, affects the spectrum of diseases that otherwise only occur in certain geographic regions. The Vienna General Hospital specializes in high-risk pregnancies. The sophisticated medical methods, such as those in neonatology, are the reason why extremely low birthweight infants and children with rare congenital diseases can survive.
- *Society:* Society’s understanding of the importance, necessity and significance of pediatric studies is constantly increasing. Nevertheless, the general population should be made even more sensitive to the importance of pediatric studies, e.g., in the form of access to research results in an easily understandable form. Medical experts also benefit by gaining experience and insight into how to use a new drug, which is then used in the particular indication and the corresponding patient sample. The transparent evidence-based approach not only promotes trust in pediatric clinical trials but also in subsequent treatment paths.
- *Technology:* Studies not only require working according to the “state of the art”, but rather working “beyond the state of the art”, which leads to scientific added value. Studies accelerate innovations that, through novel drugs, medical devices and therapeutic strategies, represent unique opportunities for improving medical care for future patients. A balance between sponsored clinical trials and competitive third-party funding for academic studies, however, is important. In Austria, the national research ratio, i.e., the research and development expenditures as a percentage of gross domestic product (GDP), amounted to 3.16 percent in the last decade [32]. This figure is above the European target (3 percent), thereby-comparable to some other European countries, such as Germany and Sweden - illustrating that Austria is an attractive study location.
- *Politics:* Medical universities have a research mission. The successful completion of studies reflects the high performance of clinical research, which in turn promotes Austria as a successful location for medicine and research. The UKKJ at the Medical University of Vienna is internationally recognized as a study center and is selected based on experience, expertise and patient population. When multi-center studies are carried out, cooperation between medical universities and hospitals is promoted at national and international level.
- *Entire economy:* The number of pediatric clinical trials is increasing. A higher number of studies usually goes hand in hand with an increased approval of pharmaceuticals and medical devices. This has immediate impact on the economy: Out of 5000–10,000 tested initial substances, only a single drug gets approved [32]. Pharmaceutical development takes about 10–12 years, with development costs of up to 2.4 billion Euros. In 2018, 84 new pharmaceuticals were approved in Europe. Between 2014 and 2018, an average of 41 new pharmaceuticals received marketing authorization in Austria [32]. Since 2007, 238 new drugs for the use on children and 39 child-friendly dosage forms have been approved [33].
  The increase in studies also requires human resources, i.e., research is an important employer for scientists, physicians, study nurses, study coordinators, clinical research associates, clinical research organizations, ethics committees, authorities, foundations, and pharmaceutical companies. Research also supports patient care with new scientific findings by uncovering direct and indirect efficiency potential.

#### 3.2.2. Task Environment

**Figure 4:**
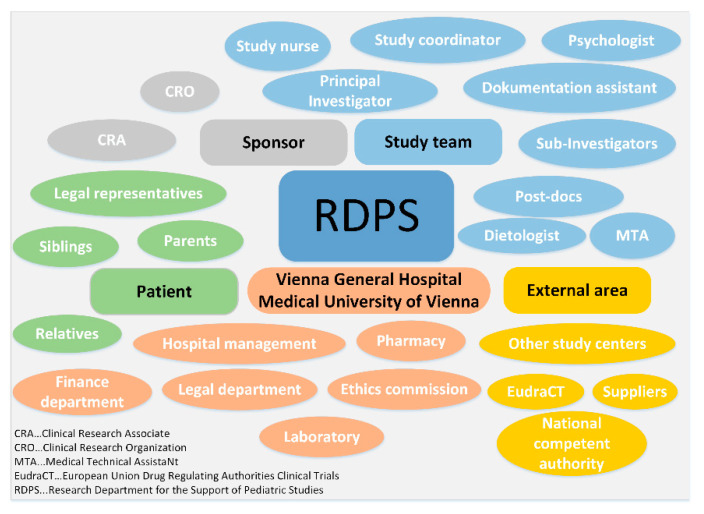
RDPS task environment - communication with stakeholders.

The task environment must be considered when implementing a RDPS, as it functions as a contact and communication interface between internal and external stakeholders. The different environments include the sponsor, the study team, the Vienna General Hospital/Medical University of Vienna, the external area and the patients. The RDPS is located at the center of these stakeholders, with the aim of uniting study-related obligations and working promptly and efficiently. Figure 4 illustrates the RDPS as an interface for the task environments, especially regarding the communication with stakeholders (Figure 4).

- *Sponsor:* The RDPS works with the sponsor at every stage of a clinical trial. Since the sponsor has many employees with different tasks, which in turn places high demands on communication, the sponsor often commissions clinical research organizations to plan, prepare and conduct a study. These clinical research organizations, also called contract research organizations (CROs), specialize in studies according to the Medicinal Products Act and the Medical Devices Act. The sponsor or clinical research organization has either its own or an external clinical research associate. The clinical research organization and the clinical research associate are therefore part of the close environment of the sponsor and thus of the RDPS. Staff fluctuations at the sponsor or the clinical research organization or a change of the clinical monitor lead to considerable information gaps. This adds to the workload and can affect the smooth running of the study. A RDPS, however, provides support in the event of sponsor-related staff fluctuations.
- *Study team:* There is a very close cooperation between the RDPS and the study team. At the stage of study initiation, there is mainly contact with the principal investigator (PI). After that, contact with the entire study team (sub-investigators, dietologist, psychologist, post-docs, medical technical assistant) is necessary, especially to plan, prepare and coordinate the visits with the study team so that all examinations can be carried out for each study visit. At the stage of study implementation, individual RDPS employees (study nurse, study coordinator, documentation assistant) are optionally members of the study team.
- *Patient:* Pediatric studies affect not only the patients, but also their families. Parents or legal representatives are involved in the entire study process. In addition, siblings and other relatives are part of the child’s environment and thus the RDPS.
- *Vienna General Hospital/Medical University of Vienna:* The Vienna General Hospital and the Medical University of Vienna are also part of the task environment of the RDPS. An important point of contact is the legal department to review the clinical trial agreement (CTA) between sponsor and the Medical University of Vienna. Although sponsors often use sample agreements that are already used for other studies at the Medical University of Vienna, adjustments to the sample agreements are often necessary before both contracting parties agree. The ethics committee, the hospital management, the finance department, the laboratory and the pharmacy are also part of the task environment.
- *External area:* The external area comprises the national competent authority, the different European drug regulating authorities, the European Medicines Agency (EMA), which administers the clinical trials database (EudraCT), other study centers, and external suppliers.

### 3.3. SWOT Analysis

Based on the results of the environmental and company analysis, we next determined the strengths/weaknesses and opportunities/threats, which were then summarized in a 4-field SWOT matrix to give decision-makers a clear overview of potential strategies for implementing a RDPS.

**Figure 5:**
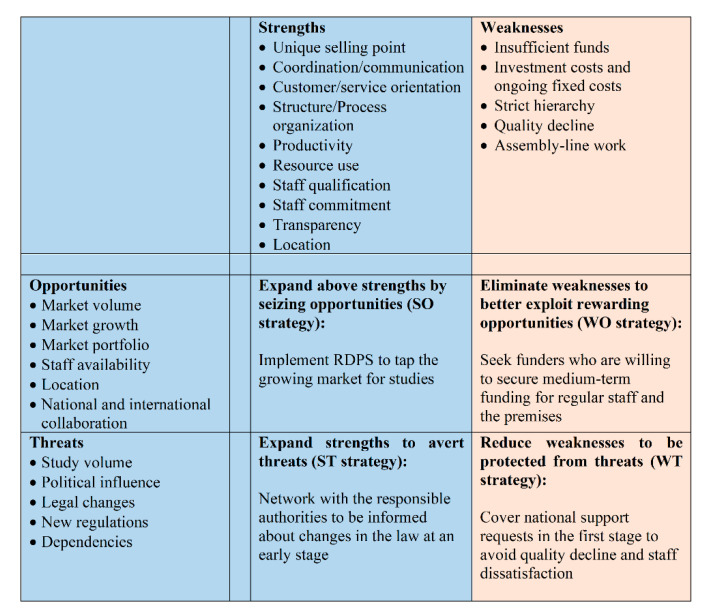
RDPS SWOT matrix.

- *Strengths:* There are several strengths when implementing a RDPS. However, due to the wide range of tasks and the variety of internal and external people involved, we refrain from discussing the strengths (and later on the weaknesses) along the management and service delivery sub-systems, as suggested in the strategic management approach.
  Since a RDPS team consists of employees, who have many years of experience in pediatric clinical studies and have contacts to experts and potential national and international partners, extensive and well-founded advice and support for studies is ensured. A high level of professionalism among team members can be assured for a team with clearly assigned tasks through relevant and continuous further education and training in connection with clinical studies. Clearly defined processes as well as clear responsibilities and work tasks of the employees not only lead to high efficiency, flexibility and service orientation, but also have a positive effect on the collaboration with cooperation partners such as pharmaceutical companies, study teams, patients and legal representatives. The own area of responsibility and the possibility of working independently promote employee satisfaction and reduce staff fluctuations, so that optimal conditions are created to keep competent employees and their know-how in the company. The many stakeholders involved in the different stages of a study also benefit from a trusted contact person at the study center.
  Professional study support is not limited to pharmaceutical studies. Hence, other studies with a high workload also benefit from the support of a RDPS. Another strength is that many different indications and studies with large and low sample sizes (e.g., rare diseases) can be carried out. A RDPS ensures that laws, regulations, and national and international guidelines are observed. The easy accessibility of the UKKJ is another advantage, not least because many patients have to undergo routine and control examinations at the UKKJ anyway.
- *Weaknesses:* The weaknesses include the high personnel costs and the hierarchical structure that is required for the organizational and internal processes for the planning and implementing of studies. If too many studies are supported at the same time, quality loss can occur. If resources are insufficient, study requests run the risk of being rejected. The standardization of processes can give employees the impression of assembly line work. Although, a high degree of standardization increases efficiency in the different stages of a study, there is a risk that the individual needs of stakeholders are not adequately addressed.
- *Opportunities:* The RDPS would be the first research department to support pediatric studies at a medical university in Austria. This pioneering role ensures a very high market coverage, since basically all types of studies (drug studies, medical device studies, non-interventional drug studies, registry studies, epidemiological studies, academic or industry sponsored studies) can be supported. Although no funding is currently planned to support basic research, a RDPS can provide support if the need arises. While the focus is on successfully setting up a RDPS in Vienna, this RDPS serves as a role model for setting up additional RDPS at other medical universities in Austria.
  The integration of the RDPS into the Medical University of Vienna opens up a wider range of opportunities for employees to undergo further training on site with regard to clinical studies. At the Medical University of Vienna, employees are offered numerous seminars free of charge, such as training on medicinal products, medicinal devices, good clinical practice, study design, pharmacovigilance, and analysis and interpretation of clinical trials. Another option for further training is participation in congresses, symposia, external workshops and seminars. There are also comprehensive training and career opportunities for young people, such as medical students, who can be recruited at an early stage of their study as documentation assistants. The time-flexible tasks of a documentation assistant can easily be combined with the six-year medical studies in Austria. When students participate in pediatric trials during their medical studies, they gain insight and experience in the field. The close collaboration with the principal investigators also gives the students the opportunity to get to know potential supervisors of theses. This offers the opportunity to complete the diploma thesis in the field of pediatrics. As graduates, they will already have extensive experience in pediatric studies, which is a competitive advantage when looking for a job, especially at a medical university.
  With regard to communication and collaboration with the many different stakeholders, the RDPS enables efficient and effective working through clearly structured processes and work instructions. Smooth processes are guaranteed by ensuring that contact persons are always available. This facilitates the work of investigators and sub-investigators in that they can primarily deal with their clinical activities.
  Existing institutions should not be seen as competitors but as potential cooperation partners. In order to be able to use synergy effects, collaborations with the KKS and the Austrian OKIDS network should be sought.
  OKIDS offers one study nurse per location. However, OKIDS is third party funded, so the availability of the OKIDS study nurse and OKIDS in general is dependent on ongoing third-party funding. The limited funding also implies that the OKIDS study nurse can only be employed 30 h per week. Given the current workload and the continued increase in drug trials, this is not enough. Additionally, since OKIDS only supports drug studies, support is limited to this type of study. The RDPS can support the OKIDS study nurse. This creates synergy effects between OKIDS and the RDPS so that the aim of increasing pediatric drug trials at the UKKJ can be achieved.
  Since the Vienna KKS concentrates exclusively on studies with adults, the KKS is not a direct competitor. We expect that the cooperation with this institution will also result in synergies. The Vienna KKS can forward any pediatric study request to the RDPS, while the RDPS can work continuously with the KKS on patient insurance. If electronic care report forms (eCRFs) or queries to pharmacovigilance are required, the KKS Vienna is a very good service provider. Since KKS charges a fee per service, KKS also benefits from a collaboration with the RDPS. A RDPS therefore not only increases the number of studies, it also increases the attractiveness of the UKKJ, the Medical University and the Vienna General Hospital as a place of study.
  With the establishment of a RDPS, there is also the possibility of flexible structuring of employment contracts. This enables nurses, who are currently working in a strictly clinical routine and who want to change careers to work as study nurses with new and challenging tasks. However, this option should not be limited to permanent staff, but should also be offered to freshly graduated nurses in order to attract highly motivated and qualified staff.
  In the medium term, the expansion of the RDPS should also be considered. With more staff and adequate training, application preparation, third party funding, budget planning, medical writing and the publication process can also be supported.
- *Threats:* Personnel costs are a high-risk factor from an external perspective as well. The key personnel (RDPS management, project manager, study coordinator, and study nurse) should hold permanent positions that are publicly funded and thus covered by the university’s budget. However, cross financing of other employees such as documentation assistants must also be guaranteed. It should also be borne in mind that it can be difficult to find competent and qualified personnel. The high level of flexibility and commitment required can also be seen as a hurdle in this regard, since the respective area of responsibility is very complex.
  The availability and cost of the premises are a further risk factor as there must be a sufficient number of rooms for employees, meetings (face-to-face, video and telephone conferences), monitoring visits and for the storage of documents and investigational medicinal products. Study documents, investigator site files and trial master files must be kept locked. In addition, all essential documents must be retained for at least 15 years after the completion of the clinical study [34].
  Although it is quite unlikely, a RDPS could be a risk factor in the form of high overhead if the core tasks of the Medical University change in such a way that research is no longer one of the core competencies. However, there is a greater likelihood of a shortage of doctors and nurses in pediatrics, as the field of pediatrics is less attractive for doctors from a financial point of view than other medical subjects and nurses have to undergo additional training.
- *SWOT Matrix:* The strengths and weaknesses as well as the opportunities and risks of the SWOT analysis must be translated into a SWOT matrix, which then forms the basis for strategy development. However, the development of strategies is the responsibility of the decision-makers at the Medical University of Vienna and was therefore not the aim of the present study. For this reason, one exemplary strategy is provided per field (Figure 5).

## 4. Discussion

The SWOT analysis provided a comprehensive overview of internal and external factors that influence the success of a RDPS. In this section, the implementation of a RDPS at the UKKJ in Vienna is discussed against the background of the expected increase in pediatric studies, the expected increase in ethical and legal norms, and the challenges in funding. Finally, we also discuss the limitations of this study.

- *Number of studies:* There are several reasons for the increase in studies at the UKKJ in Vienna. In 2013, OKIDS, the Austrian research network for pediatric drug studies, was implemented at the Department of Pediatrics and Adolescent Medicine in Vienna. As a result, more drug trials were carried out and the study teams on site acquired specialist knowledge for the successful implementation of studies. This in turn led to highly satisfied investigators and sponsors, which increased the number study requests. Another reason is the research mandate of the medical universities in Austria. At the Medical University of Vienna, just like at other medical universities, research is a cornerstone in addition to teaching and patient care. This in turn creates the incentive to conduct studies and, as a result, to acquire third party funds in order to deliver a corresponding research output. Young scientists in particular need support in this regard.
  EU Regulation (EC) No. 1901/2006 supports pediatric drug studies so that children and adolescents receive specially tested and approved drugs [3], which in itself will lead to an increase in the number of studies. In addition, Vienna has a diverse and large clientele for studies compared to other cities, which is why the UKKJ is considered as an attractive site for the study center.
- *Ethical and legal norms:* Children and adolescents are a particularly vulnerable patient population. Therefore, comprehensive guidelines and regulations must be followed, and additional monitoring carried out, which increases the need for support. We assume that the increase in ethical and legal norms observed in the past will continue in the next few years in order to expand the protection of the vulnerable patient collective. Pediatric studies are complex and resource-intensive. Investigators and study teams do not always know what needs to be considered in clinical studies. A RDPS is therefore not only available as a service provider, but also as a control body to ensure that all ethical and legal standards are observed.
  For clinical studies, the sponsor is obliged to conduct monitoring visits at the study center. The Medical University of Vienna is the sponsor for academic studies. However, monitoring is costly. Clinical research organizations or the Vienna KKS can be commissioned for this, but they are cost-intensive. Since the budget for academic studies is generally limited and a RDPS also offers monitoring, the Medical University of Vienna incurs not only additional costs when implementing a RDPS, but also savings because the monitoring can be offered by the RDPS at significantly lower costs. However, monitoring services depend on the RDPS resources, the priority, the study type, the study protocol and the sample size. For example, monitoring is not required by law for non-clinical studies, such as registry or epidemiological studies, but is recommended. If sufficient resources are available, a RDPS can also support these study types as a monitoring body.
- *Financing:* In times of limited resources, special attention is paid to the financing of a RDPS. The key staff consists of the RDPS management and at least one project manager, a study coordinator, a study nurse and a clinical research associate. Publicly funded positions should be created for key personnel, and the following options are available for financing other employees: A start-up fee can be negotiated for industry-sponsored studies. The start-up fee covers the services provided by the RDPS for the entire support right up to the initiation of the study. Charging a fee per patient is another financing option. Another source of funding is the monitoring. The sponsor can save the entire travel expenses for monitoring visits by commissioning a RDPS monitor. This is a great financial advantage for the sponsor, as well as for the RDPS, which means that funds are re-acquired and some of it can be passed on to the RDPS. However, services provided by a study nurse, a study coordinator or a documentation assistant are available exclusively for a single study and have to be budgeted accordingly.
- *Limitations:* As a typical strategic management tool, SWOT analysis was originally designed for private profit-oriented organizations. However, in view of the growing challenges in healthcare, including the aging of the population, the rapid technological change, and the increase in healthcare expenditure while financial resources are increasingly limited, strategic planning has also been used by healthcare organizations. For a discussion of the pros and cons of strategic planning in healthcare organizations see Rodríguez Perera and Peiró [35]. As indicated by Chermack and Kasshanna, if adequately used, SWOT analysis provides support in deriving appropriate strategies to meet overall goals by exploiting the organization’s strengths and the environmental opportunities and mitigating the organization’s weaknesses and environmental threats [36]. However, as with any other tool, pitfalls have also been identified in the use of SWOT analysis, not least because the user has a considerable leeway in performing a SWOT analysis. The authors argued that a “critical flaw in the development of SWOT analysis as a solid and reliable strategic tool is a lack of research” [36], thereby criticizing that theory building for SWOT analysis is based on empirical applications only. They also offered protocols to address various pitfalls in the uses of SWOT analysis. Pitfalls include, among others, the use of SWOT analysis as justification for decisions already made; the negligence of the close relationship between the results of the SWOT analysis and the subsequently derived strategy; the failure to link the results of the company analysis with those of the environmental analysis; and deriving strategies before all strategic options have been identified. For the comprehensive list of pitfalls see, e.g., Koch [37] and Kearns [38].

The use of SWOT analysis regarding the implementation of a RDPS has proven to be an easy-to-use and flexible tool to analyze both the status quo and the dynamics of the company and its environment. The SWOT analysis must be regularly adjusted and updated to take account of changes in the company and business environment. The survey effort, however, is enormous to get a reliable database. If data is missing or data is inaccessible, this leads to information gaps. The possibility of reducing complexity, increasing transparency, visualizing internal and external influencing factors is certainly a great advantage, which is why the SWOT analysis was considered a suitable method for evaluating the implementation of RDPS.

However, further work is required based on the list of items collected by any SWOT analysis. To derive an actual strategy, it might be necessary to score individual items, since it is unlikely that all items collected are equally important. In order to avoid a high degree of subjectivity, it is advisable not to have the scoring carried out by a single person. The same applies to the derivation of actual strategies and action plans. As with any other tool, the outcome of SWOT analysis heavily depends on the care with which this tool is used. If the SWOT analysis is updated at regular intervals, dynamic developments can be taken into account in the form of updated strategies. If, however, SWOT analysis is performed judiciously, as the literature shows, it can be used in a variety of ways to support decision-makers.

## 5. Conclusions

The SWOT analysis shows that a RDPS offers a unique concept to support any type of pediatric study. A key advantage of implementing a RDPS is that the study nurse’s activities do not merge with those of a study coordinator. Both a study coordinator and a study nurse are essential for the success of a study and should have clear tasks. A RDPS enables conducting multicenter clinical studies at an internationally recognized quality level.

The implementation of a RDPS leads to a centralization of pediatric studies. The focus is on pooling tasks and concentrating specialist knowledge, achieving synergy potential, ensuring standardized processes, avoiding inefficiencies in relation to duplication of work, which promotes flexibility, efficiency, professionalism and innovation. The RDPS enables tailor-made advice and support for different types of studies.

According to the upcoming patient/parent and public involvement designs, this research unit can communicate efficiently with stakeholders, users and companies, since the interdisciplinary engagement is fostered from the very beginning according to a system-theoretic approach [11,12]. Specific cost-consequence analyses can easily be conducted, as health-economic knowledge is included from the very beginning of the strategic planning phase.
